# BAP1 inactivation promotes lactate production by leveraging the subcellular localization of LDHA in melanoma

**DOI:** 10.1038/s41420-024-02250-6

**Published:** 2024-11-26

**Authors:** Guopei Zheng, Jiahao Shi, Qian Li, Xiaoliang Jin, Yan Fang, Zhe Zhang, Qin Cao, Lili Zhu, Jianfeng Shen

**Affiliations:** 1 Department of Ophthalmology, Ninth People’s Hospital, Shanghai Jiao Tong University School of Medicine, Shanghai, 200025 China; 2 Shanghai Key Laboratory of Orbital Diseases and Ocular Oncology, Shanghai, China; 3 Institute of Translational Medicine, National Facility for Translational Medicine, Shanghai Jiao Tong University, Shanghai, China; 4 Bio-X Institutes, Key Laboratory for the Genetics of Developmental and Neuropsychiatric Disorders, Ministry of Education, Shanghai Jiao Tong University, Shanghai, China; 5 Songjiang Research Institute and Songjiang Hospital, Shanghai Jiao Tong University School of Medicine, Shanghai, China

**Keywords:** Eye cancer, Cell growth

## Abstract

BRCA1-associated protein 1 (BAP1) acts as a tumor suppressor and can affect the cell cycle, tumor immunity, and cellular metabolism through multiple pathways. In melanoma, BAP1 mutations promote tumor cell glycolysis, leading to increased lactate production. The tumor microenvironment with high lactate levels is often associated with immunosuppression and tumor progression. The inhibitory effect of BAP1 on glycolysis has been found in a variety of tumors, but the specific mechanism by which BAP1 inhibits lactate production still needs to be elucidated. In this study, we show that BAP1 can interact directly with lactate dehydrogenase (LDHA), causing LDHA to accumulate in the nucleus. Conversely, BAP1 deletion leads to the accumulation of LDHA in the cytoplasm, catalyzing the production of lactate from pyruvate that results in increased lactate levels inside and outside the cell. By elucidating the interaction between BAP1 and LDHA and the subsequent effects on lactate production in melanoma cells, this work provides insights into the mechanism of BAP1-mediated metabolic regulation. Furthermore, it may provide novel directions for the clinical treatment of BAP1-mutant melanoma.

## Highlights

- BAP1 loss promotes an acidic tumor microenvironment through upregulation of glucose metabolism.
- BAP1 interacts with LDHA, but does not affect its enzymatic function.
- BAP1 supports LDHA nuclear translocation to inhibit its glucose metabolic function.

## Introduction

BRCA1-associated protein 1 (BAP1) was originally identified as an interacting protein of Breast cancer type 1 susceptibility protein (BRCA1). BAP1 is mainly localized in the nucleus and exerts tumor suppressive effects by binding to the RING finger domain of BRCA1 [1]. As a ubiquitin carboxy-terminal hydrolase, BAP1 regulates post-translational modifications of a variety of proteins through its deubiquitinating enzymatic activity, which can control DNA damage repair, cell metabolism, cell cycle, immune regulation, and other processes [2, 3]. BAP1 is an important tumor suppressor, and inactivating mutations of BAP1 have been identified in a variety of malignancies. Among them, BAP1 mutation rates are high in uveal melanoma (UVM) and malignant mesothelioma (MESO), and these mutations have also been found to varying degrees in tumors such as skin cutaneous melanoma (SKCM) and kidney renal clear cell carcinoma (KIRC) [4, 5]. Germline mutations in BAP1 can lead to BAP1 tumor susceptibility syndrome, where patients often develop multiple malignancies including UVM at a younger age and with a higher probability [4].

Numerous studies have shown that BAP1 plays an important role in metabolic regulation. Metabolic reprogramming is an important feature of malignant tumors and a contributing factor to tumor development. Activation of oncogenes or inactivation of tumor suppressor genes can alter the metabolic pathways of cells and promote tumor formation and progression [6]. Loss of function of BAP1 leads to elevated levels of anaerobic glycolysis in fibroblasts, and these cells become more susceptible to carcinogenesis due to the Warburg effect [7]. In UVM, BAP1 mutations may lead to metabolic heterogeneity with the expression levels of glycolysis and oxidative phosphorylation (OXPHOS) [8]. Therefore, the design of therapeutic approaches to target metabolic heterogeneity can be a promising direction to treat BAP1 mutant tumors. Inactivation of BAP1 alters the metabolic status of different tissues and regulates various metabolic pathways such as lipids, nucleotides, and calcium ions [9, 10], which have a broad role in tumor development. In melanoma, BAP1 deficiency significantly elevated anaerobic glycolysis levels, leading to more lactate production in tumor cells [11]. However, the specific mechanism of BAP1-mediated regulation of lactate production requires further exploration.

High levels of aerobic glycolysis and lactate production are characteristic metabolic alterations observed in most cancer cells. A microenvironment with high lactate is associated with tumor aggressiveness and correlates with both melanoma progression and treatment [12–14]. High level of lactate impairs the function of immune cells and creates an immunosuppressive microenvironment that favors tumor development [15, 16]. Lactate dehydrogenase (LDH) is a key enzyme to reduce pyruvate in the cytoplasm with NADH to generate lactate and produce NAD+, which is essential for the continuity of glycolysis [17]. Lactate dehydrogenase A (LDHA) is widely distributed in a variety of tissues and is the major LDH isoform that catalyzes lactate production [18, 19]. LDHA expression is elevated in a variety of malignancies and is thought to be a metabolic adaptation of tumor cell glycolysis [20–23]. LDHA is associated with T-cell immune activation [24], and high LDH expression levels correlate with low patient survival rates, clinical staging, and disease recurrence [25–28]. Inhibition of LDH activity has antiproliferative effects on cancer cells, and there has been recent renewed interest in LDH as an anticancer drug target [29, 30]. In addition to the classical intracytoplasmic lactate catalytic activity, LDHA displays nuclear translocation in several types of tumor cells [31]. LDHA in the nucleus has non-classical enzymatic activity, which catalyzes the generation of hydroxybutyrate from pyruvate and is associated with oxidative stress and activation of the WNT signaling pathway [32–34]. Tumor cells often display differences in acidity inside and outside the cell [35], and the differences are related to transport proteins such as Monocarboxylic acid transporters (MCT) and a variety of other factors [36–40]. Because the nuclear pore complex strictly controls the shuttling of proteins between the cytoplasm and nucleus, it remains unclear how LDHA lacking the nuclear localization signal (NLS) sequence undergoes nuclear translocation. It is possible that the presence of atypical NLS signals or interacting proteins in LDHA promotes its nuclear translocation.

In this study, we have found that BAP1 interacts with LDHA. Importantly, BAP1 is required to constrain the subcellular localization of LDHA in nucleus, thereby reducing the amount of cytoplasmic LDHA and inhibiting lactate production.

## Results

### Glycolysis levels are increased in BAP1-knockdown cells

**Fig. 1:**
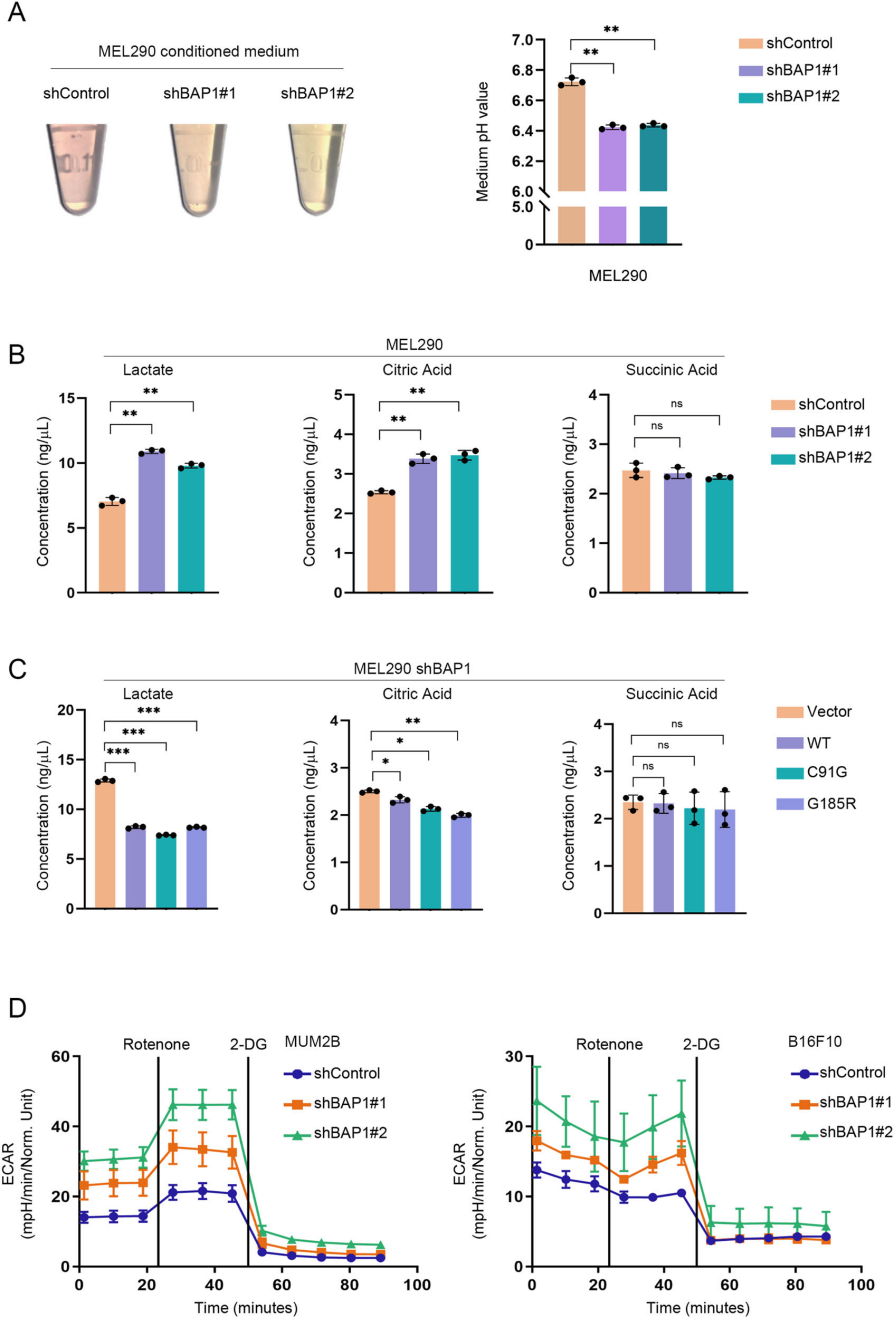
Glycolysis levels are increased in BAP1 knockdown cells. **A** Cell culture medium pH decreased after BAP1 knockdown. Samples are cell culture medium of BAP1 knockdown group and control group in UVM cell line (MEL290). **B** Changes in lactic acid, citric acid and succinic acid levels after BAP1 knockdown. Samples are cell culture medium from BAP1 knockdown group and control group, and values were determined using the corresponding kits respectively. **C** Changes in lactic acid, citric acid and succinic acid content after reintroduction with BAP1. Exogenous BAP1 (wild type, enzyme active site mutants C91G and G185R) was complemented in the BAP1 knockdown MEL290 cell line, and the extracellular lactate levels were measured after the complementation. **D** Increase in cellular glycolytic capacity after BAP1 knockdown. Glycolytic capacity of BAP1 knockdown and control groups in MUM2B and B16F10 was detected using a hippocampal extracellular flux analyzer and analyzed by Agilent Seahorse XF reporter generator. *p < 0.05, **<0.01, ***<0.001, and no significance (ns).

We found that the culture medium of the MEL290 cells with BAP1 knockdown was more acidic than that of control cells during cell culture (Fig. 1A, Supplementary Fig. 1A). Because lactate is a major metabolite of tumor cell glycolysis, we first examined the lactate levels in the culture medium. When BAP1 was knocked down, MEL290 cells produced more lactic acid (Fig. 1B). The same alterations were detected in KIRC and SKCM cell lines (Supplementary Fig. 1B, C). Considering the tricarboxylic acid cycle (TCA), the level of citric acid was modestly increased when BAP1 was knocked down, but the succinic acid level was not changed (Fig. 1B), suggesting BAP1 only had limited effects on TCA cycle. After complementation with exogenous wild-type (WT) BAP1 and its mutant forms (C91G, G185R) in BAP1-depleted MEL290 cells, the lactic and citric but not succinic acid levels were decreased (Fig. 1C, Supplementary Fig. 1D). We also used the endpoint cell number as a normalization to the lactate level, and found consistent results as observed using initial normalization (Supplementary Fig. 1E). We examined the glycolytic capacity of UVM (MUM2B) and SKCM (B16F10) cell lines using a hippocampal extracellular flux analyzer. The data suggested that the BAP1 knockdown cell lines had higher levels of glycolysis with increased glycolytic proton efflux rate (glycoPER) and extracellular acidification rate (ECAR), which were consistent with the lactate production analysis (Fig. 1D, Supplementary Fig. 1F, G).

### BAP1 and LDHA have protein-to-protein interactions

**Fig. 2:**
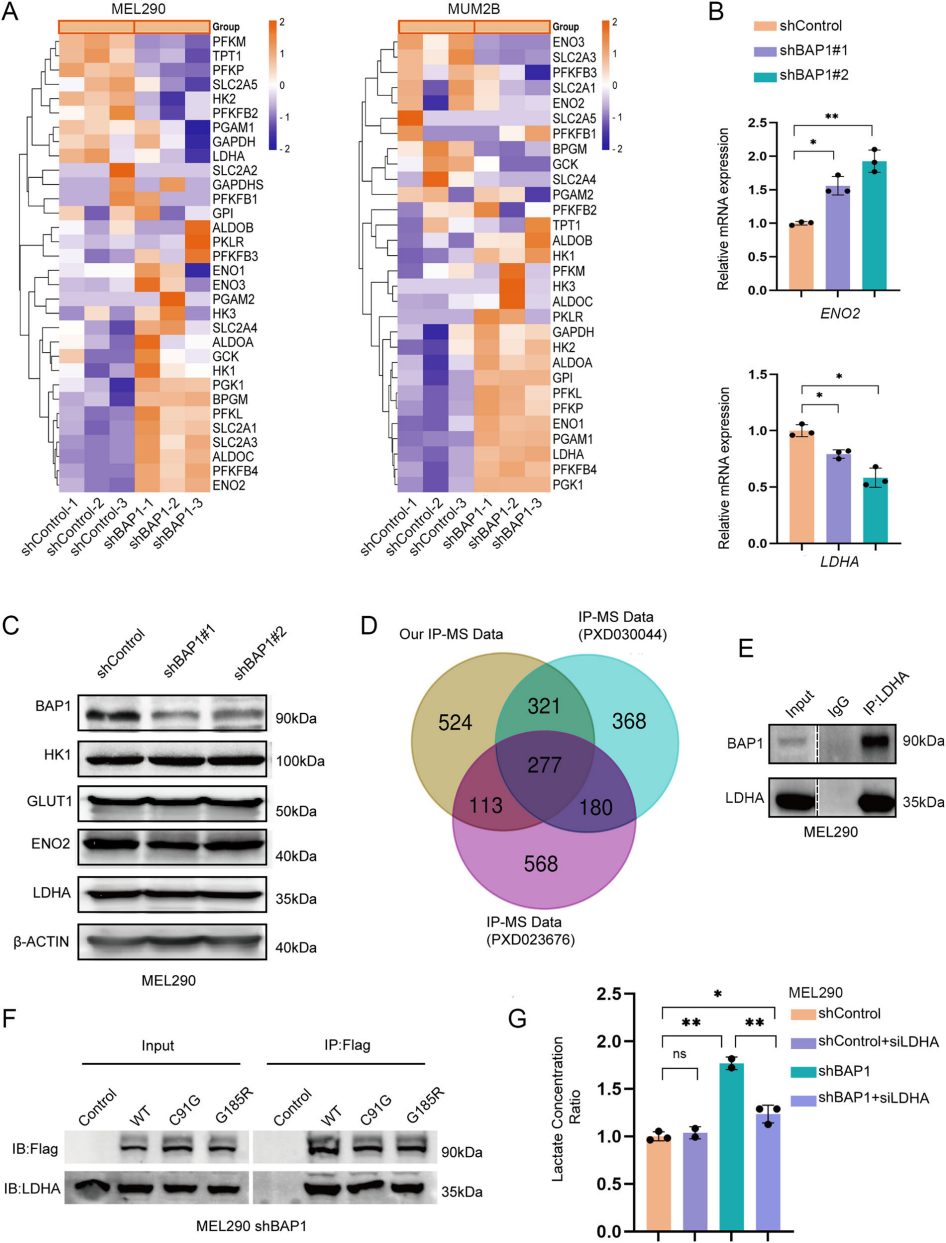
BAP1 and LDHA have protein-to-protein interactions. **A** Heat map showing the mean expression values of genes related to glucose metabolism, data from RNA-seq data of BAP1 knockdown group and control group in MEL290, MUM2B cell lines. **B** Fluorescence quantitative PCR was performed on differentially expressed genes, and the samples were MEL290 cell lines. **C** Western blot of differentially expressed genes to detect protein expression levels in the BAP1 knockdown and control groups of MEL290 cell lines. **D** Immunoprecipitation of BAP1 was performed on the MEL290 cell line, and protein characterization was performed using triple quadrupole tandem mass spectrometry and compared with published mass spectrometry data (PXD030044, PXD023676). **E** Immunoprecipitation of LDHA was used to detect its interaction with endogenous BAP1. **F** Immunoprecipitation of exogenous BAP1 was used to detect its interaction with LDHA. **G** Simultaneous knockdown of LDHA in the BAP1 knockdown and control groups, respectively, and detection of changes in extracellular lactate levels. *p < 0.05, **<0.01, ***<0.001, and no significance (ns).

We sought to explore the specific mechanisms underlying the effects of BAP1 on glycolysis. Firstly, we analyzed the effect of BAP1 on the transcriptional level of glycolytic pathway. By analyzing the differential gene expression with BAP1 knockdown in MEL290 cells, we found that the expression levels of some glucose metabolism-related genes, such as ENO2 (Enolase 2) and SLC2A1 (Solute Carrier Family 2 Member 1), were upregulated, while some genes, such as LDHA, were downregulated (Fig. 2A). These results were further confirmed by real-time quantitative PCR using the MEL290 cell line (Fig. 2B). These findings also differed in various cell lines such as B16F10 cell line, suggesting that the effect of BAP1 on glycolytic pathway gene expression may be inconsistent (Supplementary Fig. 2A). Subsequently, we examined the expression levels of glycolysis related proteins such as LDHA, and the results showed no significant changes in the protein levels of glycolytic enzymes. (Fig. 2C). We then reasoned that the change of mRNA level may not eventually lead to the change of protein amount because many factors such as translational and post-translational modifiers could affect the protein level. Therefore, these data suggest that BAP1 does not affect glycolysis in UVM by regulating the expression of glycolytic enzymes, we need to consider other possible mechanisms. We then examined the profile of interacting proteins of BAP1 using immunoprecipitation combined with mass spectrometry (IP-MS). By comparing our IP-MS results with other published mass spectrometry data, we found that multiple glycolytic enzymes may interact with BAP1 (Fig. 2D, Supplementary Fig. 2B). We further performed immunoprecipitation experiments on several candidates and found that LDHA can interact with BAP1 (Fig. 2E, Supplementary Fig. 2C). We also performed immunoprecipitation experiments in cell lines supplemented with wild-type and enzymatically inactive mutant BAP1, and interactions between LDHA and BAP1 were detected in each group (Fig. 2F). This suggests that the interaction between these two proteins may not be dependent on BAP1 enzymatic activity. Therefore, we knocked down LDHA in MEL290 cells (Supplementary Fig. 2D). Interestingly, LDHA depletion significantly rescued the lactate levels in BAP1 knockdown cells (Fig. 2G), indicating that BAP1 exerted an inhibitory effect on the lactate producing function of LDHA.

### The enzymatic activity of LDHA is not directly inhibited by BAP1 protein

**Fig. 3:**
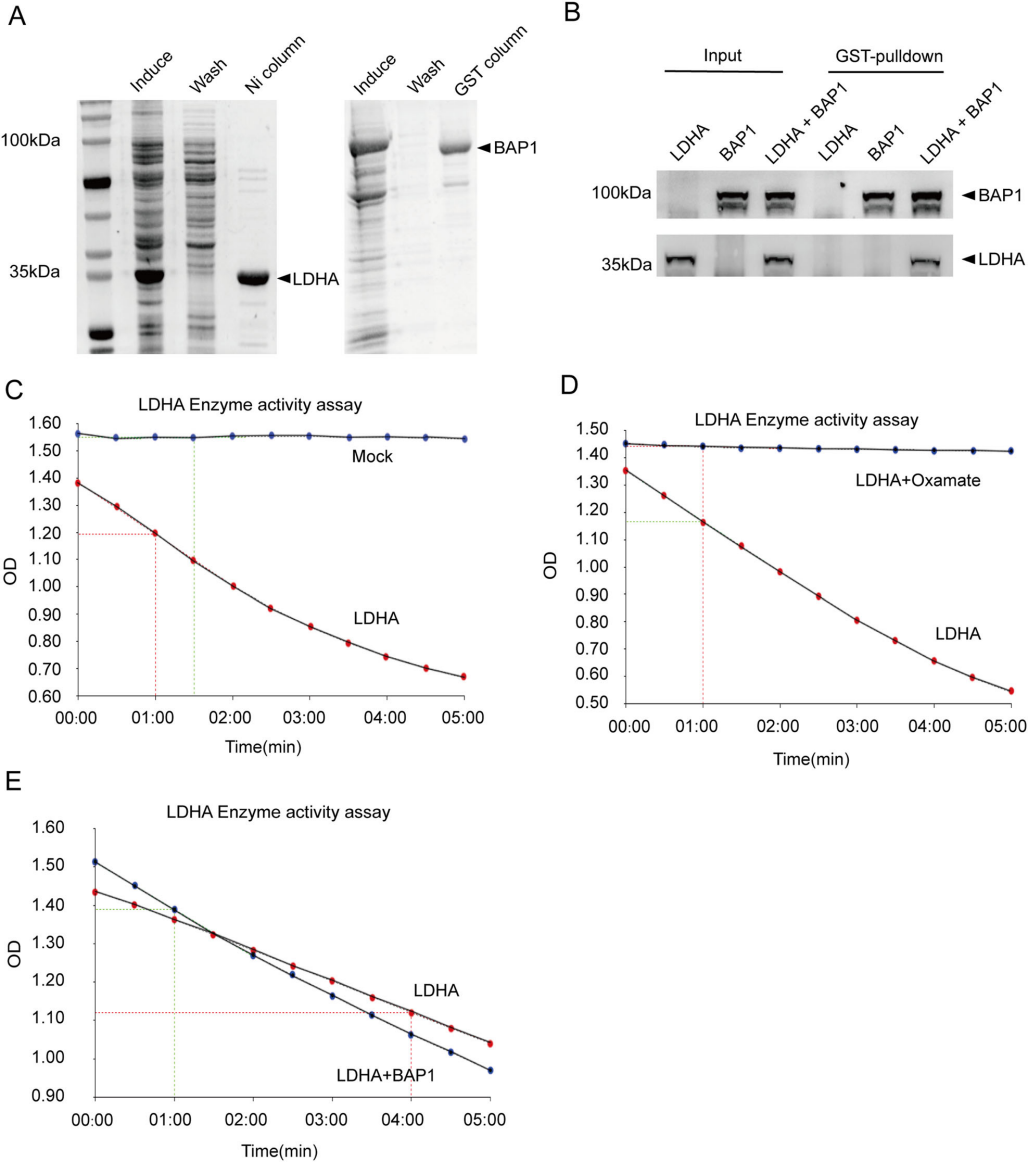
The enzymatic activity of LDHA is not directly inhibited by BAP1 protein. **A** Human-derived full-length LDHA and full-length BAP1 were expressed in E. coli, and the proteins were purified using Ni column affinity chromatography and GST column affinity chromatography, respectively. **B** Examination of the direct interaction between GST-BAP1 and LDHA in vitro by GST-Pulldown. **C**–**E** The purified LDHA proteins were used for kinetic assays of enzyme activity, and the absorbance was measured at 340 nm to determine the amount of NADH. **C** Only substrate was added in the Control group, and LDHA was added in the LDHA group. **D** The enzymatic activity of LDHA was completely inhibited after the addition of 50 mM oxamate to LDHA. **E** The enzymatic activity of LDHA was not significantly changed after the addition of an equal amount of BAP1 protein to LDHA.

To investigate whether BAP1 directly inhibits the enzymatic activity of LDHA by binding to LDHA, we generated full-length BAP1 and LDHA expression constructs, then expressed them in *Escherichia coli* system to obtain purified proteins by affinity chromatography (Fig. 3A). Through GST-pulldown assay, we confirmed that BAP1 has direct interaction with LDHA in vitro (Fig. 3B, Supplementary Fig. 2E). Because LDHA catalyzes the generation of lactate from pyruvate while changing NADH to NAD+, we examined the enzymatic activity using the substrate NADH content as the standard (Fig. 3C). The purified LDHA protein showed stable enzymatic activity, but this was subsequently significantly inhibited after the addition of oxamate (Fig. 3D). Finally, the ability of LDHA to catalyze lactate production was not significantly altered after the addition of protein BAP1 to LDHA (Fig. 3E). These results suggested that BAP1 does not inhibit the catalytic function of LDHA through its direct binding.

### BAP1 inhibits lactate production through increased nuclear localization of LDHA

**Fig. 4:**
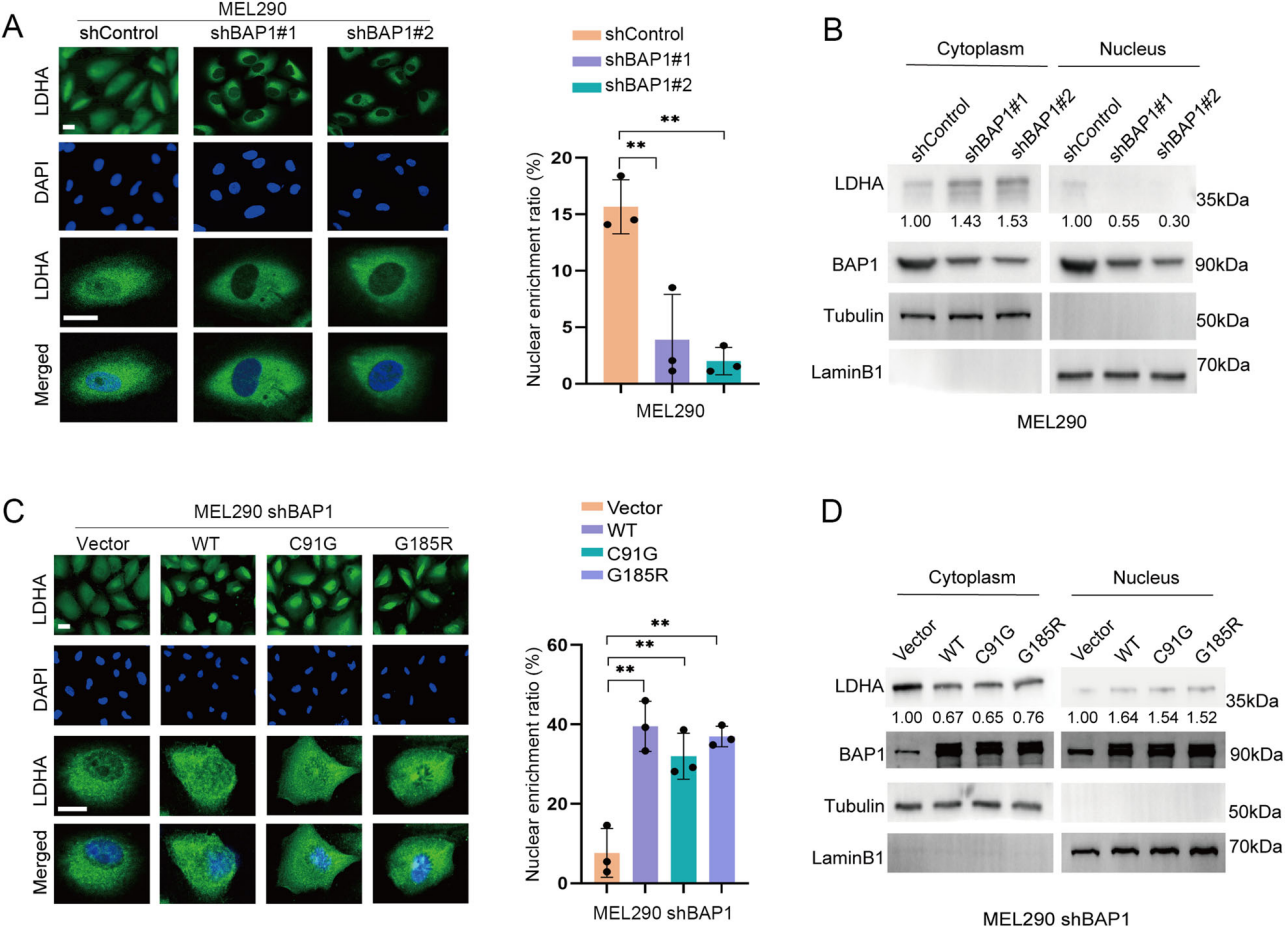
BAP1 affects lactate production by regulating the subcellular localization of LDHA. **A** Immunofluorescence staining of BAP1 knockdown cell lines. Reduced nuclear localization of LDHA in the BAP1 knockdown group. Each datapoint represents the mean value of nuclear enrichment ratio of each condition from three experimental replicates. Scale bar is 20 µm. **B** Western blotting for nucleus and cytoplasm separation of BAP1 knockdown cell lines. **C** Immunofluorescence staining of BAP1 reintroduction cell lines. The nuclear translocation of LDHA was significantly increased after complementation back to exogenous BAP1. Quantification of nuclear localization of LDHA cells as a percentage of total cell numbers, respectively. Scale bar is 20 µm. **D** Western blotting for nucleus and cytoplasm separation of BAP1 reintroduction cell lines.

To investigate the specific mechanism through which BAP1 affects LDHA function, we performed immunofluorescence staining for LDHA in BAP1 knockdown and reintroduction MEL290 cells. Immunofluorescence results showed that nuclear translocation of LDHA was significantly reduced after knockdown of BAP1 (~19% to ~8%) (Fig. 4A). We isolated the nucleus and cytoplasm extracts to examine the distribution of LDHA, and found that the amount of nuclear LDHA was decreased by BAP1 knockdown (Fig. 4B). In contrast, after reintroduction with exogenous BAP1, LDHA significantly aggregated in the nucleus. The nuclear translocation of LDHA was calculated to increase from ~15% to ~46% (Fig. 4C), indicating that the interaction between BAP1 and LDHA affected its subcellular localization. Subsequently, the fractionation and western blotting also supported this conclusion (Fig. 4D). When BAP1 was ablated, LDHA was more likely to localize in the cytoplasm to increase the lactate production rates. Comparable degree of LDHA nuclear translocation was observed when BAP1 inactive mutants (C91G, G185R) were introduced, indicating that the subcellular localization of LDHA was independent of the deubiquitinating enzymatic activity of BAP1 (Fig. 4C). Confocal microscopy images show that LDHA had significant intranuclear aggregation when BAP1 was overexpressed (Fig. 4C). We knocked down LDHA and reconstructed MEL290 cells with exogenous wildtype (LDHA-WT) and LDHA with nuclear localization signal (NLS) (LDHA-NLS). Notably, LDHA-NLS cells produced less lactate compared with that of LDHA-WT cells (Supplementary Fig. 2F). In line with previous reports [32], our results demonstrated that cytoplasm was the preferential site for LDHA to produce lactate and change of the subcellular localization indeed affected lactate production.

### Full-length BAP1 is required for nuclear localization of LDHA

**Fig. 5:**
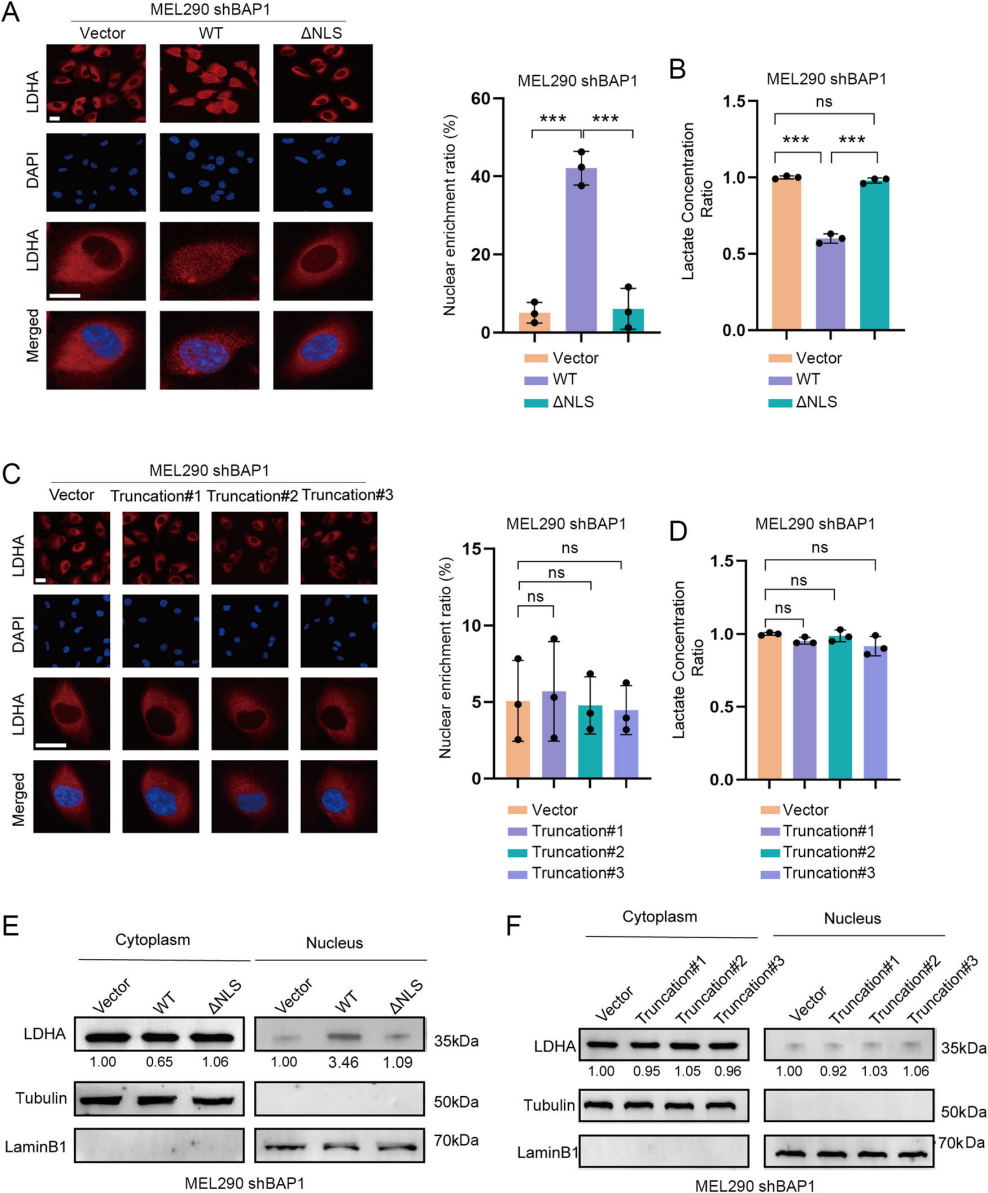
Full-length BAP1 is required for nuclear localization of LDHA. **A** Complementation of exogenous NLS-deleting BAP1 mutant (ΔNLS) and wild-type BAP1 (WT) in BAP1-knockdown MEL290 cell lines and performing Immunofluorescence staining. Each datapoint represents the mean value of nuclear enrichment ratio of each condition from three experimental replicates. Scale bar is 20 µm. **B** Measuring lactate content in cell lines with BAP1-WT and BAP1-ΔNLS. **C** Complementation of exogenous BAP1 truncations in BAP1-knockdown MEL290 cell lines and performing Immunofluorescence staining. Intranuclear localization of LDHA in cells did not change significantly after reintroduction of BAP1 truncation mutants. Scale bar is 20 µm. **D** Measuring lactate content in cell lines with BAP1 truncation mutants. **E**, **F** LDHA was detected at different sites by Western blotting after isolating the nucleus and cytoplasm.

We then reasoned that the binding of BAP1 to LDHA was critical to mediate the translocation of LDHA to the nucleus since BAP1 but not LDHA harbored the NLS sequence. To test, we constructed the NLS-deleting BAP1 mutant (ΔNLS, amino acid residues 1-699) and found this mutant still interacted with LDHA (Supplementary Fig. 3A). reintroduction of wildtype but not ΔNLS BAP1 significantly increased the amount of nuclear LDHA as shown in immunofluorescence (IF) staining (Fig. 5A), and dramatically decreased the lactate level (Fig. 5B). We constructed truncation mutants of BAP1 (1-300aa, 301-550aa and 551-711aa) and performed reintroduction experiments in BAP1-knockdown MEL290 cells. We found that these truncation mutants lost the binding ability with LDHA (Supplementary Fig. 3A) and failed to change the subcellular localization of LDHA (Fig. 5C). Notably, these BAP1 mutants cannot rescue the phenotype of lactate production (Fig. 5D), suggesting that full-length BAP1 was required to maintain the interaction between BAP1 and LDHA. The western blotting on fractionation extracts also showed that NLS-deleting and truncation mutants of BAP1 did not increase the nuclear localization of LDHA (Fig. 5E, F), confirming the results of IF staining. These results demonstrated that both interaction with LDHA and nuclear translocation of BAP1 were essential for BAP1 to carry LDHA into the nucleus. Therefore, enzyme active site mutants of BAP1 (C91G, G185R) that did not affect BAP1-LDHA interaction and BAP1 nuclear translocation were still capable of regulating the subcellular localization of LDHA.

Because the BAP1 truncations are unable to interact with LDHA, and NLS-deleting BAP1 is unable to carry LDHA into the nucleus, neither of which affects the intracellular localization of LDHA. Enzyme active site mutants of BAP1 (C91G, G185R) do not affect the interaction with LDHA and are therefore able to regulate the localization of LDHA. This suggests that full-length BAP1 is required for nuclear localization of LDHA.

### Glycolytic pathway is upregulated in BAP1-knockdown melanoma cells

According to the experimental flow diagram show (Supplementary Fig. 4A), after inoculation of B16F10 tumor cells into mice, we performed transcriptomic sequencing analysis (RNA-Seq) on mouse tumors to verify the specific effect of BAP1 on melanoma glycolysis in vivo. Gene ontology analysis of the differentially expressed genes between the BAP1 knockdown and control groups showed that the expression levels of metabolic processes (GO:0008152) were upregulated after BAP1 knockdown (Supplementary Fig. 4B). We analyzed the UVM in The Cancer Genome Atlas (TCGA) database and found that multiple cellular metabolic pathways, including glycolysis, were enriched in UVM cases with low expression of BAP1 (Supplementary Fig. 4C). A similar manifestation was observed in UVM with BAP1 mutation (mostly truncation) (Supplementary Fig. 4C). We further performed a gene set enrichment analysis on glycolysis genes and found that glycolysis genes were enriched in UVM with low BAP1 expression levels compared with normal BAP1 expression (Supplementary Fig. 4D). In the analysis of the UVM data in TCGA, it was found that the tricarboxylic acid cycle pathway was also upregulated when BAP1 was lowly expressed, which may be related to the increased glycolytic products (Supplementary Fig. 3B). Enrichment of glycolytic genes was also seen in BAP1-mutant tumors compared with wild-type BAP1 tumors (Supplementary Fig. 4D). The correlation analysis revealed that BAP1 repressed glycolytic gene expression in UVM, but its correlation was weak (Supplementary Fig. 3C), suggesting that BAP1 may affect glycolysis through multiple mechanisms, and transcriptional changes caused by BAP1 on glycolysis may not be significant at the lactate level because of molecular cascade reactions or the influence of other proteins. Survival analysis of the TCGA UVM dataset showed that low BAP1 expression levels were detrimental to patient survival rates (Supplementary Fig. 3D), while LDHA expression was not associated with overall survival of patients (Supplementary Fig. 3E).

We induced oxidative stress in cells using Doxorubicin and measured alterations in cell count employing the Cell Count Kit-8. Our experiments revealed that BAP1 knockdown inhibited the proliferation of MEL290 cells and rendered them insensitive to Doxorubicin (Supplementary Fig. 5A, B). In our experiments, the knockdown of LDHA yielded outcomes that were comparable to those observed in cells with BAP1 knockdown. However, the knockdown of LDHA did not rescue the effects of BAP1 knockdown on cell proliferation, with or without Doxorubicin treatment (Supplementary Fig. 5A, B). These observations suggest that BAP1 and LDHA might exert effects on cell proliferation and oxidative stress signaling via multiple, potentially independent mechanisms, which are likely not primarily mediated through the regulation of lactate production.

All these results demonstrated that BAP1 affects UVM glycolysis by modulating the subcellular localization of LDHA. This also explains the inconsistency between the level of glycolysis and the expression of glycolytic enzymes. LDHA localized in the cytoplasm catalyzes the conversion of pyruvate to lactate, meanwhile producing NAD+ to maintain the glycolytic process. Through protein-protein interaction, BAP1 mediates the translocation of LDHA from cytoplasm to nucleus, where LDHA no longer produces lactate. In BAP1-deficient cells, the nuclear retention of LDHA was unleashed and the subsequent cytoplasmic accumulation occurred, resulting in the elevation of both glycolysis rate and lactate production (Supplementary Fig. 5C).

## Discussion

BAP1 plays an important role in processes associated with DNA damage repair, cell metabolism, cell cycle, and immune regulation [2, 3]. In the present study, we found that BAP1 interacts with LDHA, which supports the nuclear translocation of LDHA. When BAP1 is absent, LDHA is localized in the cytoplasm. Because LDHA is a key lactate-producing enzyme, higher cytoplasmic levels of LDHA increase the cellular lactate levels and produces the NAD+ required for the glycolytic process. This study further explains the specific mechanism by which a mutation in BAP1 can lead to both increased glycolysis levels and lactate production in tumor cells.

LDHA undergoes nuclear translocation in a variety of tumors including cervical tumors [32]. Nuclear-localized LDHA has non-classical enzymatic activity by catalyzing the production of hydroxybutyrate or hydroxyglutarate [32, 41]. However, LDHA itself does not contain the NLS region, so the exact mechanism controlling its nuclear translocation is unknown. BAP1, which does have an NLS region, mainly functions in the nucleus. Therefore, it can be speculated that BAP1 could facilitate the translocation of LDHA into the nucleus through its interaction with BAP1. However, the full details of this mechanism merit further investigation.

As a well-established tumor suppressor, BAP1 is mutated across a variety of human cancers, and patients with BAP1 mutations often exhibit worse prognosis [4, 42]. BAP1 promotes the development and recruitment of T cells and is essential to maintain the anti-tumor immunogenicity [43]. Since the change of lactate levels has a profound impact on immune cell behavior [15, 16], it remains to be illustrated that, to what extent, BAP1 loss could cause immune evasion of tumor cells through the modulation of acidity in tumor microenvironment. LDH inhibitors have been developed for cancer therapy, however its clinical applicability is still under evaluation because of the associated non-specific tissue toxicity and pharmacokinetics [44, 45]. Intriguingly, biomarker-guided application of LDH inhibitors may be a practical way. In this regard, it is reported that LDH inhibitors resulted in marked reduction of lactate production, with no serious adverse effects in patients with congenital deficiency of LDH5 [46]. These findings raise the possibility that LDH may serve as a therapeutic target in patients with BAP1 deficiency.

In conclusion, this study identifies a novel mechanism by which BAP1 can regulate glycolysis in tumor cells. BAP1 interacts with and maintains the nuclear localization of LDHA. BAP1 perturbation leads to the accumulation of LDHA in cytoplasm to promote the production of lactate and NAD+. By elucidating the mechanism of lactate regulation by BAP1, our work provides new directions for the development of targeted metabolic therapies for BAP1-deficient tumors.

## Materials and methods

### Cell culture

MEL290, MUM2B, B16F10, and RENCA cell lines were purchased from the China National Collection of Authenticated Cell Cultures. MEL290, MUM2B, B16F10, and RENCA were cultured in RPMI Medium 1640 (#C11875500BT, Gibco) containing 10% FBS (#FSP500, ExCellBio) and 293T was maintained in DMEM supplemented with 10% FBS. All cell culture media were supplemented with 1% penicillin/streptomycin (#15140-122, Gibco) and maintained at 37°C in a humidified 5% CO2 environment. Cell lines were regularly tested for mycoplasma.

### RNA interference (RNAi)

siRNA was purchased from GenePharma, transfected using Lipofectamine 3000 (#L3000001, Thermo Fisher Scientific) transfection reagent according to the manufacturer’s instructions, and cells were cultured in medium containing the transfection mixture for 48 h before harvesting. siRNA sequence: BAP1#1 GAGGCUGAGAUUGCAAACUTT, BAP1#2 CGGCCUUUCUAGACAAUCATT, BAP1#3 GCAGCUGAUAAGAGAGUAACATT, LDHA#1 CUGGCAAAGACUAUAAUGUTT, LDHA#2 CCAGUUUUCCACCAUGAUUATT, LDHA#3 CGGUUGCAAUCUGGAUUCATT, Negative Control UUCUCCGAACGUGUCACGUTT.

### Transfection and virus packaging

Four shRNA sequences targeting BAP1 were cloned into the pLKO.1-puro vector. The targeting sequences for BAP1 were: Targeting human BAP1 (pLKO.1-shBAP1#1: CCGGCGTGGAAGATTTCGGTCAACTCGAGTTGACACCGAAATCTTCCACGTTTTTG; pLKO.1-shBAP1#2: CCGGATCATGCCACGGTCCCAACTACTCGAGTAGTTGGGACCGTGGCATGATTTTTTG). Targeting mouse Bap1 (pLKO.1-shBap1#3: CCGGCCCTCAGTATTACCATGTCTTCTCGAGAAGACATGGTAATACTGAGGGTTTTTG; pLKO.1-shBap1#4: CCGGCCCTCAGTATTACCATGTCTTCTCGAGAAGACATGGTAATACTGAGGGTTTTTG). Wild-type and deubiquitinating enzyme active site mutants (C91G, G185R) BAP1 were cloned into pHAGE-puro vector for overexpression of BAP1. Plasmid transfection was performed using PolyJet DNA in vitro transfection reagent (SignaGen, #SL100688) according to the instructions provided by the manufacturer. After lentiviral packaging using HKE293T cells, the corresponding cell lines were infected using the collected lentivirus and cultured with 2 μg/mL puromycin for 3 days for screening. Truncation mutants (1–300aa, 301–550aa and 551–711aa) and NLS-deleting mutant (1–699aa) BAP1 were cloned into pHAGE-puro vector for overexpression of BAP1. Wild-type LDHA and LDHA with nuclear localization signal (NLS) was cloned into pHAGE-puro vector for overexpression of LDHA. Plasmid transfection was performed using Polyjet. Mixing 1 μg plasmid with 3 μL Polyjet evenly, and incubating in cell culture dishes for 48 h.

### Animal experiments

B16F10 cells from the BAP1 knockdown and control groups were injected subcutaneously into the legs using C57BL/6 mice, and approximately 5 × 10^6^ tumor cells were used per mouse. Tumor growth was observed daily, and mice were euthanized at day 20 of tumor cell inoculation. The tumors were removed and subsequent experiments were performed. All experimental procedures were approved by the Institutional Animal Care and Use Committee of Shanghai Jiao Tong University.

### RNA extraction and real-time PCR

RNA was extracted from 1 × 10^6^ cultured cells using the RNA extraction kit (#B0004DP, EZB) according to the manufacturer’s instructions. mRNA was reverse transcribed to cDNA using the reverse transcription reagent (#R323, Vazyme) according to the manufacturer’s instructions. PCR was performed in a Real-Time system (CFX384, Bio-Rad) using SYBR Green PCR reagent (#Q711, Vazyme) according to the manufacturer’s instructions. PCR amplification of the housekeeping gene ACTB was performed for each sample as a control for sample loading and normalization of different samples. PCR primer sequences were as follows: BAP1:GGTTTCAGCCCTGAGAGCAA (forward), GGGCCTGGCATGGCTATTAT (reverse); ACTB: CACTGTCGAGTCGCGTCC (forward), TCATCCATGGCGAACTGGTG (reverse); ENO2: CTCGAGGAGATCCCAGCCA (forward), AGCCCGGAAAAGACCTTTGG (reverse); SLC2A1: CTGGCATCAACGCTGTCTTC (forward), GTTGACGATACCGGAGCCAA (reverse); SLC2A3: CTGTAGGACCCGAGGAACAC (forward), GATGGGGTCACCTTCGTTG (reverse); SLC2A4: CCTCGGCAGCGAGTGAC (forward), AGCTCTGTTCAATCACCTTCTGT (reverse); PFKM: GGTTTGGAAGCCTCTCCTCC (forward), GGGTCATGATCCACTCTTGTAGT (reverse); LDHA: GTCCAAGATGGCAACCCTCA (forward), CTCATCCGCCAAGTCCTTCA (reverse); LDHB: AAAGGCTACACCAACTGGGC (forward), GCCGTACATTCCCTTCACCA (reverse); ALDOA: TCCTGTGCCAGGAAAGCAC (forward), GCGATGTCAGACAGCTCCTT (reverse); ALDOC: AGAGGAAAAGTGAGCTGTGCT (forward), GGCATGATGACAGTTGTCTATACTC (reverse); HK1: AGCCGCCATTGAAACGGATA (forward), GCATACGTGCTGGACCGATA (reverse); GPI:: TGTCTACGAACACGGCCAAA (forward), TGGCTGACCACAGCGAATAG (reverse).

### RNA sequencing

After obtaining sample RNA, RNA integrity was analyzed using agarose gel electrophoresis and RNA purity was checked using a NanoPhotometer spectrophotometer. RNA libraries were created using the NEBNext® UltraTM RNA Library Prep Kit (Illumina), and the quality of the libraries was checked using an Agilent 2100 bioanalyzer and RT-PCR. Once library quality testing was completed, the different libraries were sequenced by Illumina according to the effective concentration and target requirements, and 150 bp paired-end reads were generated. Data quality was checked and data analysis was performed.

### Western blotting

Cell samples were lysed using buffer containing β-ME and urea. Supernatants were collected after centrifugation and protein concentrations were quantified using the Bicinchoninic Acid (BCA) Protein Assay Kit (Pierce). Equal amounts of protein (30 µg) were separated by 10% SDS-PAGE after denaturation and transferred to PVDF membranes (Millipore). The membranes were blocked with 5% skim milk (Bio-Rad). Incubate with primary antibody, followed by secondary antibody with fluorescent moiety and detected in ChemiDoc MP imaging system (BIO-RAD). The primary antibodies used were: BAP1 (#D1W9B,1:1000) from Cell Signaling Technology; Hexokinase (#19662-1, 1:1000), LDHA (#66287-1, 1:1000), TXNIP (#18243-1, 1:1000), SLC2A1 (#21829-1, 1:1000), ALDOA (#11217-1, 1:1000) LaminB1 (#12987-1, 1:1000), Alpha-Tubulin (#11224-1, 1:1000), Flag tag (#66008-4, 1:1000) from ProteinTech; β-actin (#AC026, 1:10000) from Abclonal. The secondary antibody used was: Alexa Fluor Plus800 (#A32735, 1:10000) from Invitrogen.

### Flux experiment

The XFe24 analyzer (Agilent Technologies) was used to measure ECAR. glycolytic stress test kit (#103020-100) was purchased from Agilent (Santa Clara, CA) and injected with compounds at concentrations of rotenone (1 µM) and 2-DG (50 mM). Analysis was performed by Agilent Seahorse XF Report Generator.

### Immunoprecipitation-mass spectrometry (IP-MS)

Cell samples were lysed using buffer containing NP-40, the supernatant was collected after centrifugation, and proteins were separated using BAP1 antibody and magnetic beads (#70024, Cell Signaling Technology). The obtained protein samples were characterized using nanoliter liquid chromatography with quadrupole orbitrap mass spectrometer (Easy nLC1200/Q Exactive plus, Thermo Fisher Scientific).

### Immunofluorescence and confocal microscopy

MEL290 cell lines were fixed to slides, immunofluorescence staining was performed using LDHA primary antibody and secondary fluorescent antibody (as described in Western Blot), and the subcellular localization of LDHA was observed using a fluorescence scanner (Pannoramic midi, 3DHistech) and cells were counted with the corresponding software slide-viewer. We used 3DHISTECH software to scan the immunofluorescence images of cells and to calculate the nuclear enrichment ratio (proportion of cells with nuclear LDHA staining). Briefly, the number of cells with nuclear LDHA was counted and divided by the total cell number to determine the nuclear enrichment ratio. Cells were further observed using a laser confocal microscope (TCS SP8 STED 3X, Leica).

### Lactic, citric and succinic acids test

Adjusted the initial seeding number to obtain comparable amounts of cells in each conditions according to the cell growth rate, or normalized by endpoint cell number, the conditional medium was collected at 48 h and test for lactic, citric and succinic acid content. Protein was filtered using an ultrafiltration tube (#MRCPRT010, Merck). Lactic acid, citric acid and succinic acid were detected using separate kits: Lactic Acid Assay kit (#MAK064, Merck), Citric Acid Content Assay Kit (#BC2155, Solarbio), Succinic Acid Content Assay Kit (#S486325, Aladdin). The values were measured in a Synergy Multimode Reader (BioTek).

### Protein purification

The full-length human LDHA cDNA reverse-transcribed from mRNA was inserted into pET-24a (+) vector that a His tag was added in 5’ terminals of LDHA. BL21 (Sangon Biotech) were transformed with the LDHA-inserted pET vector, and the cells were cultured in LB broth medium at 37° C until OD600 became 06–0.8. Then, the temperature was reduced to 16°C and isopropyl-β-D-thiogalactoside (IPTG) was added to the medium until incubation for 12 h. The cells were collected by centrifugation at 4 °C and broken by ultrasound in balance buffer (20 mM Tris-HCl, 500 mM NaCl, 1 mM TCEP, pH 7.4). The crude extract was collected using centrifugation for 20 min at 4 °C at 30,000 × *g* and purified on Ni-affinity chromatography with a linear gradient of Imidazole between 0 mM and 500 mM. The fractions were examined by sodium dodecylsulfate polyacrylamide gel electrophoresis (SDS-PAGE) with a 10% (w/v) acrylamide gel. The full-length human BAP1 in pGEX-4T1 with a GST tag was purified using same protocol.

### GST pulldown

Configure a buffer of 25 mM Tris-HCl, 150 mM NaCl, pH 7.4. The GST-BAP1 obtained from in vitro purification was replaced with LDHA in the above buffer, and 1 mg of GST-BAP1 was added to the GST beads (#20562ES25, Yeasen) and incubated on a shaker for 2 h (4 °C). The beads were washed three times using the same buffer, and 0.5 mg LDHA was added and incubated on a shaker for 2 h (4 °C). The beads were washed again three times, the beads were eluted using 20 mM GSH (#G105426, Aladdin), and the samples were subjected to SDS-PAGE.

### LDHA enzyme activity assay

Configure a buffer of 25 mM Tris-HCl, 150 mM NaCl, pH 7.4 and dilute the purified LDHA to 4 ng/L. Configure a substrate mixture of 1.6 mM β-NADH (#N8129, Sigma) and 4 mM sodium pyruvate (#11360070, Gibco) in a 96-well Clear Plate Add 50 µL LDHA and 50 µL mixed substrate and detect kinetics at 340 nm absorbance. A control group with the addition of 50 mM oxamate or 4 ng/L BAP1 was also set up.

### Cell proliferation and death assay

MEL290 cells were inoculated into 96-well plates, 1 × 10^3^ /well, with 5 replicates of each cell line. Doxorubicin (10 µg/mL, 3.5 h) was added after cell attachment, and 100 µL of CCK8 (#SBCCK8, Sharebio) was added to each well after drug treatment, and the reaction was carried out at 37 °C for 1 h. The absorbance was detected at 450 nm. Cell proliferation levels were assayed at 24 h, 48 h, and 72 h using the same method.

### Statistical analysis

Statistical analysis of the relevant data was performed using GraphPad Prism 8 software. An unpaired t-test was used to generate p values, with p < 0.05 indicating statistical significance.

## Supplementary information

Supplementary legends

Supplementary Figures

WB RAW DATA

## Data Availability

The datasets generated during and/or analyzed during the current study are available from the corresponding author on reasonable request.
