# Supplementary material for: BAP1 inactivation promotes lactate production by leveraging the subcellular localization of LDHA in melanoma
Source: Cell Death Discov. 2024 Nov 26;10:483. doi: 10.1038/s41420-024-02250-6 (PMC11589756; doi:10.1038/s41420-024-02250-6)
Supplement: Supplementary file 1 — Supplementary legends [file 41420_2024_2250_MOESM1_ESM.docx]

**Supplementary figure 1**

**A**. Stable knockdown of BAP1 in the MEL290 cell line. **B**. Stable knockdown of BAP1 in the B16F10 and RENCA cell lines. **C**. Changes in lactate levels after BAP1 knockdown were detected separately inB16F10 and RENCA cell lines. **D**. Exogenous BAP1 (wild type, enzyme active site mutants C91G and G185R) was complemented in the BAP1 knockdown MEL290 cell line. **E**. Lactate levels in cell lines with BAP1 knockdown or reintroduction were examined by normalizing with endpoint cell number. **F**. Detection of PER (Oxygen Consumption Rate) and OCR (oxygen consumption rate) of MUM2B. **G**. Detection of PER (Oxygen Consumption Rate) and OCR (oxygen consumption rate) of B16F10.

**Supplementary figure 2**

**A**. Detection of mRNA levels of some glycolytic genes in BAP1-KD MEL290 using RT-PCR. **B**. Examination of the overlap between the reciprocal protein profile of BAP1 and the glycolytic gene set (GO:0006110, GO:0061615). **C**. Immunoprecipitation of LDHA in BAP1 knockdown MEL290 to detect the interaction between LDHA and exogenous BAP1. **D**. Validation of LDHA protein expression using siRNA knockdown of LDHA. **E**. Examination of the direct interaction between GST-BAP1 and LDHA in vitro by GST-Pulldown. **F**. LDHA-knockdown MEL290 cells were complemented with wild-type LDHA (LDHA-WT) and LDHA with NLS sequences (LDHA-NLS). Lactate levels increased after Complementation with exogenous LDHA.

**Supplementary figure 3**

**A.** Constructing truncation mutants of BAP1 (Truncation#1-3: 1-300aa, 301-550aa and 551-711aa) and NLS-deleting BAP1 mutant (ΔNLS, 1-699aa). Performing the reintroduction experiments in BAP1-knockdown MEL290 cells and detecting the interaction between BAP1 mutants and LDHA through Co-IP. **B**. Enrichment analysis of UVM data of TCGA, tricarboxylic acid cycle (TCA) genes were enriched in the BAP1 low expression group. **C**. Analysis of BAP1 correlation with glycolytic gene set (GO:0006110) in TCGA's UVM databases. **D-E**. Analysis of the relationship between BAP1 or LDHA expression status and patients' overall survival using data from UVM in TCGA.

**Supplementary figure 4**

**A**. Experimental flow chart. Establishment of BAP1 stable knockdown cell lines, mouse tumor inoculation and RNA-seq analysis. **B**. Detection of genes differentially expressed after BAP1 knockdown using transcriptome sequencing and identification of altered biochemical pathways by GO analysis of Metascape. **C**. Use of UVM data from TCGA, grouped according to BAP1 expression or mutation. Smaller FDR values were significant. **D**. Categorical analysis of the UVM data in TCGA, the glycolytic gene set was enriched in the BAP1 low expression group and the mutation group.

**Supplementary figure 5**

**A**. Growth curves of MEL290 cells, detected using CCK8, longitudinal ordinate is the absorbance at 450 nm. **B**. MEL290 cells were treated with Doxorubicin and changes in cell number were detected using CCK8. **C**. Summary Diagram. *p<0.05, **<0.01, ***<0.001, and no significance (ns).
