# Supplementary material for: BAP1 inactivation promotes lactate production by leveraging the subcellular localization of LDHA in melanoma
Source: Cell Death Discov. 2024 Nov 26;10:483. doi: 10.1038/s41420-024-02250-6 (PMC11589756; doi:10.1038/s41420-024-02250-6)

Supplementary Figure 1

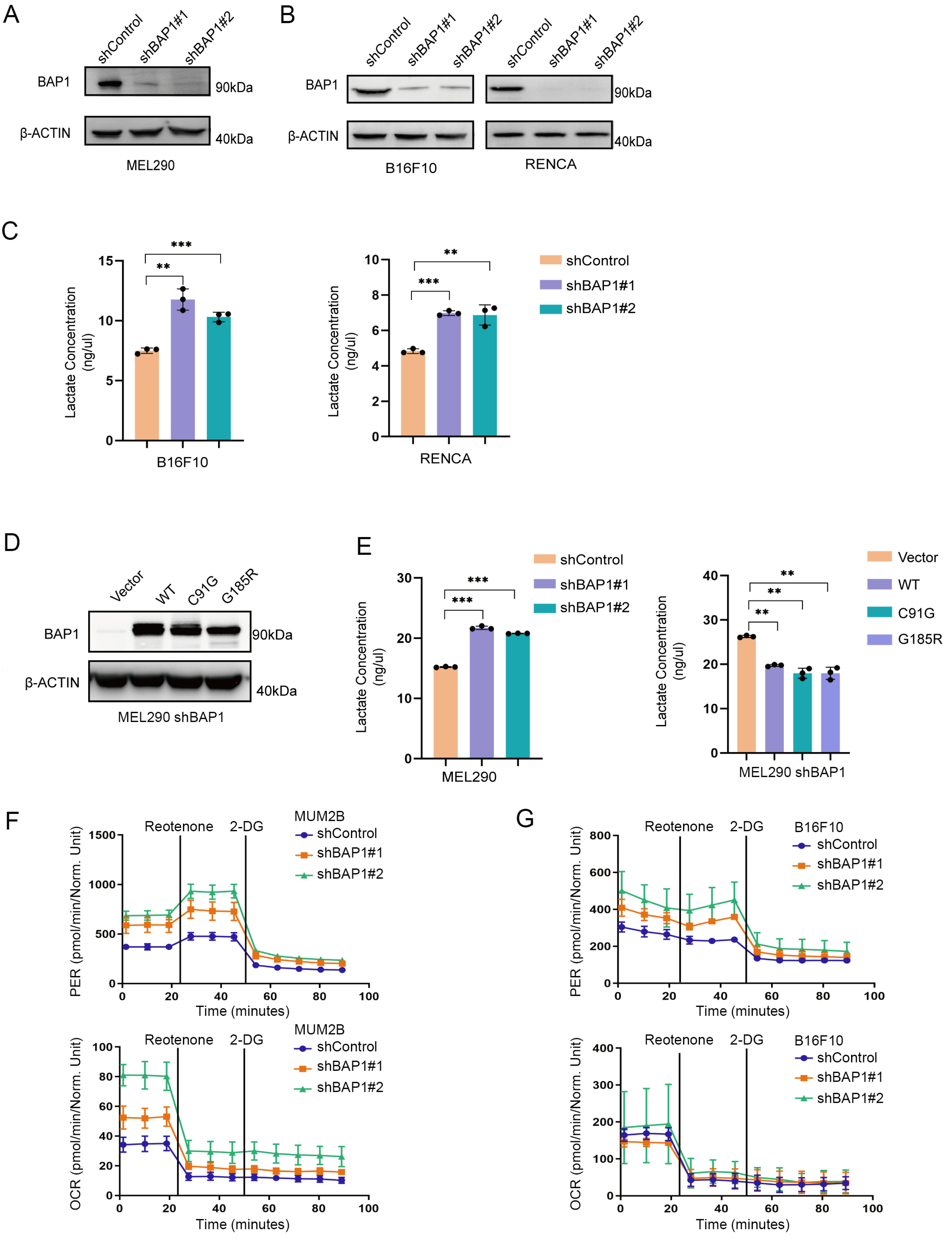

Supplementary Figure 2

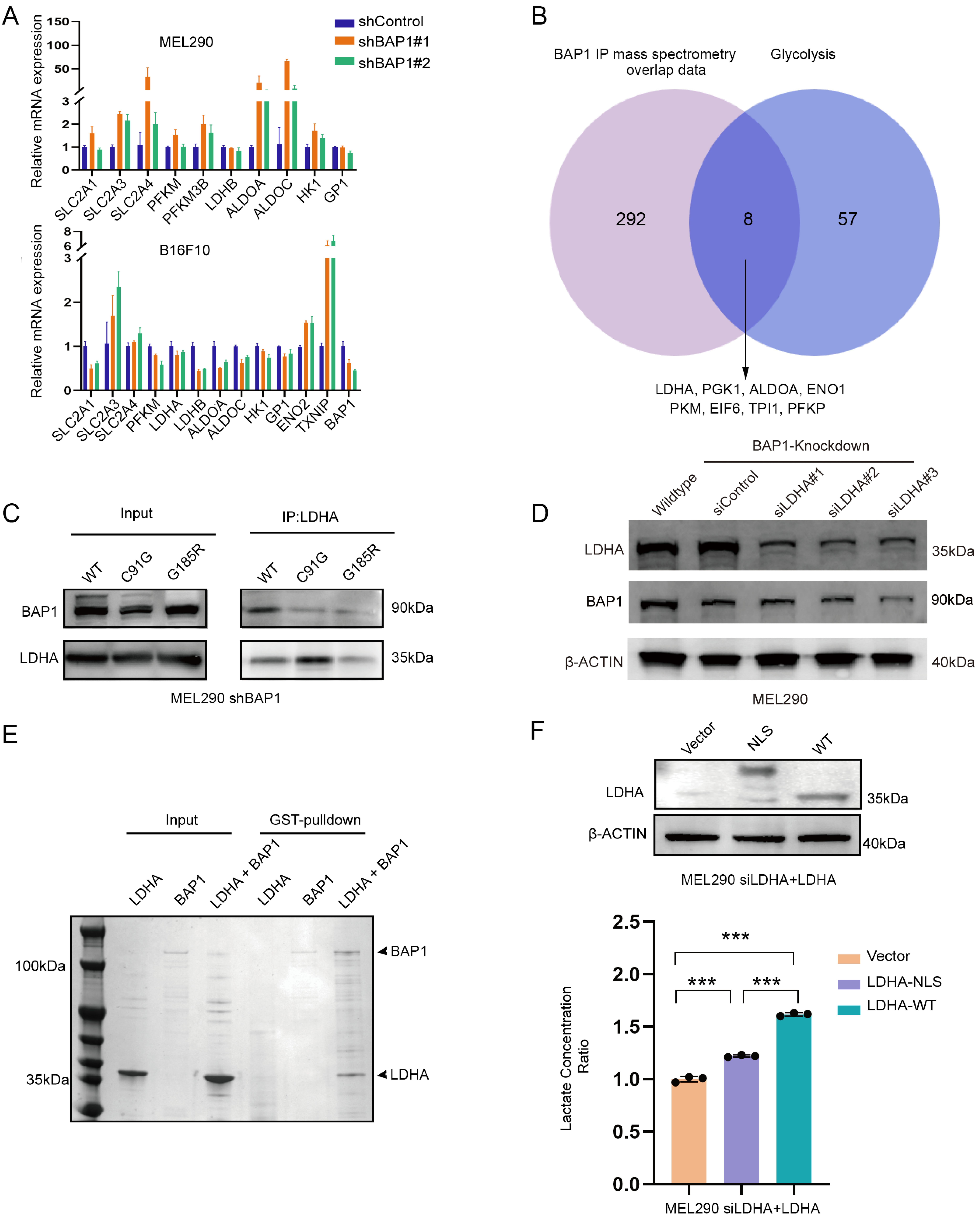

Supplementary Figure 3

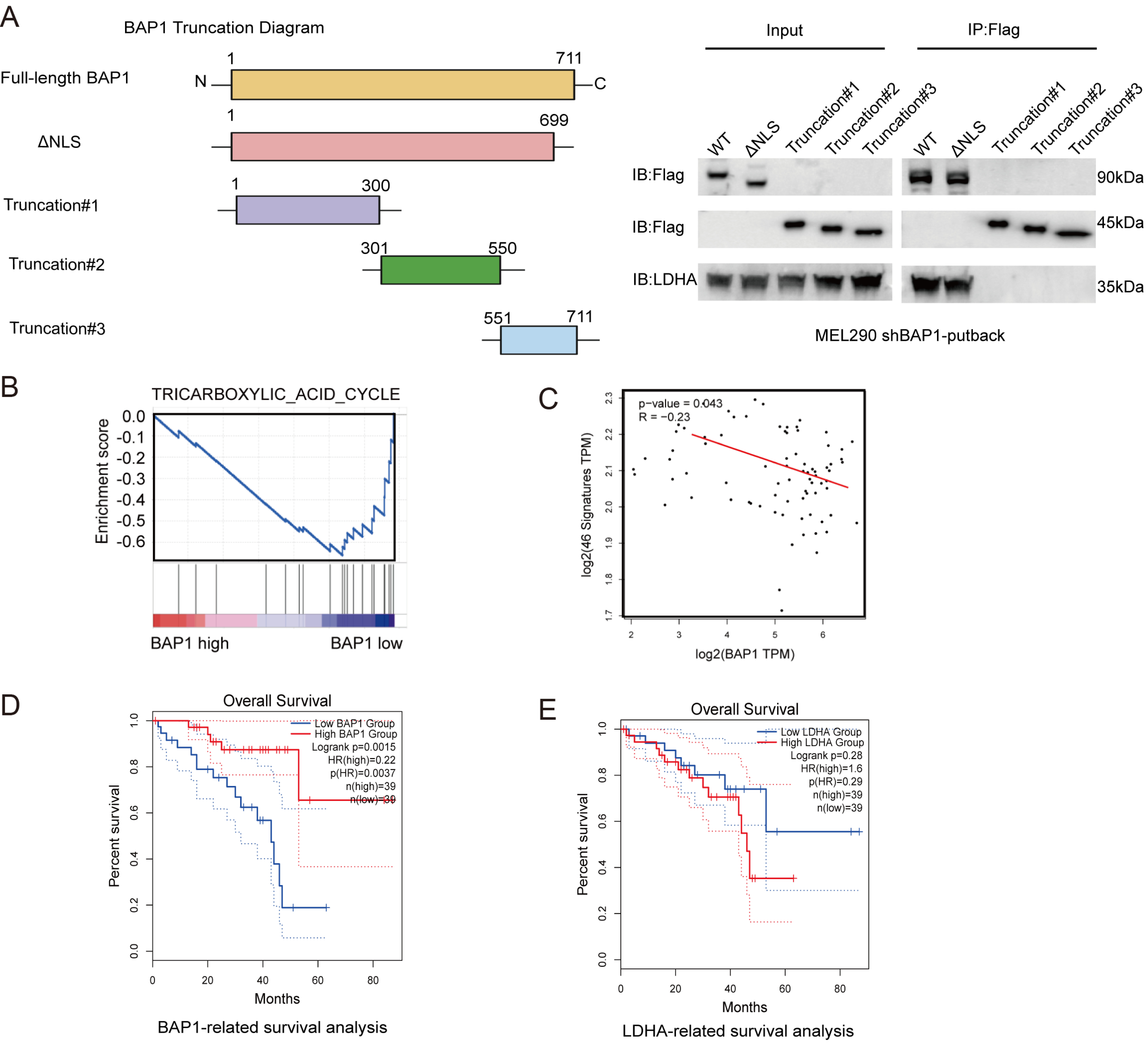

Supplementary Figure 4

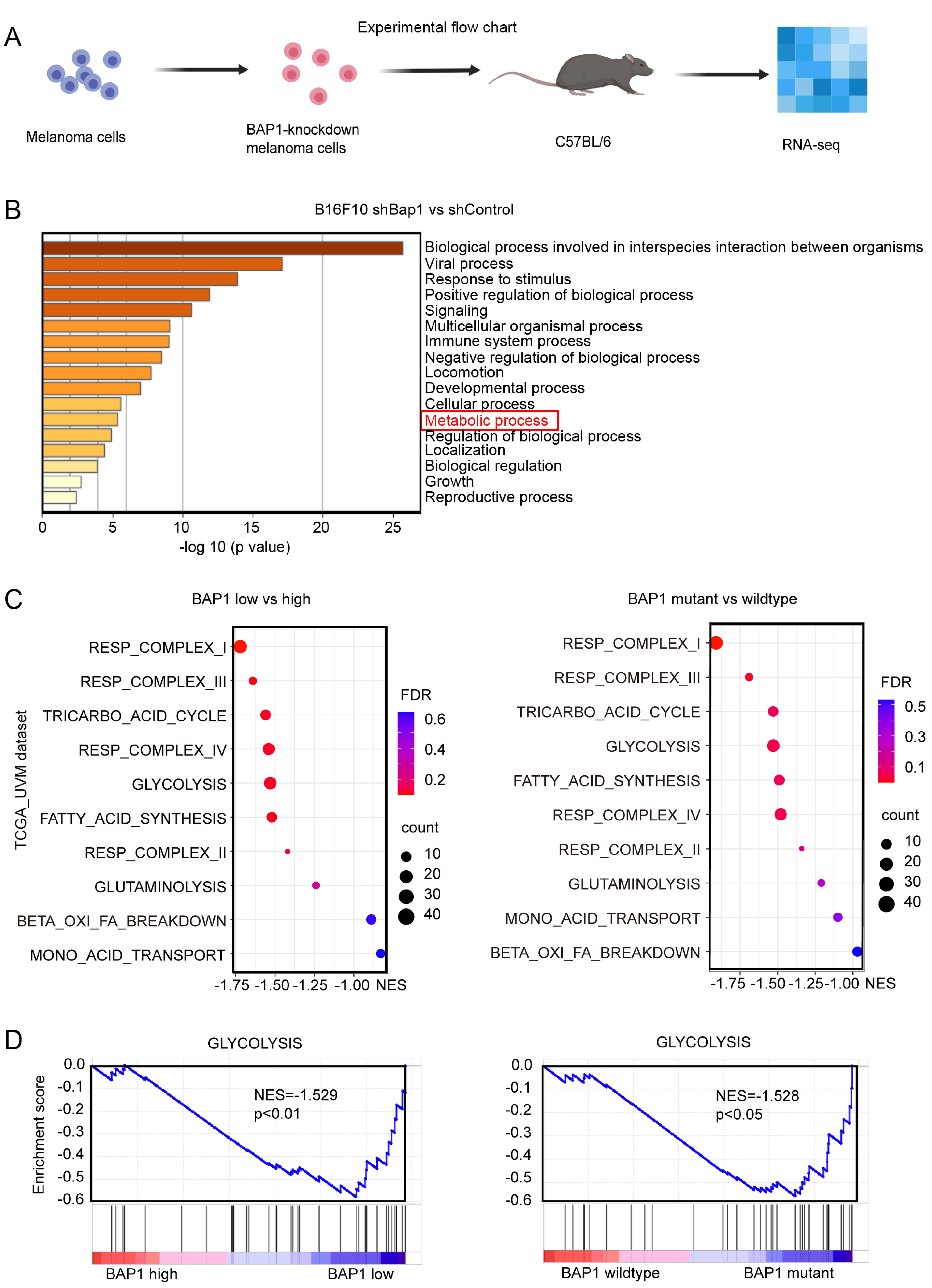

Supplementary Figure 5

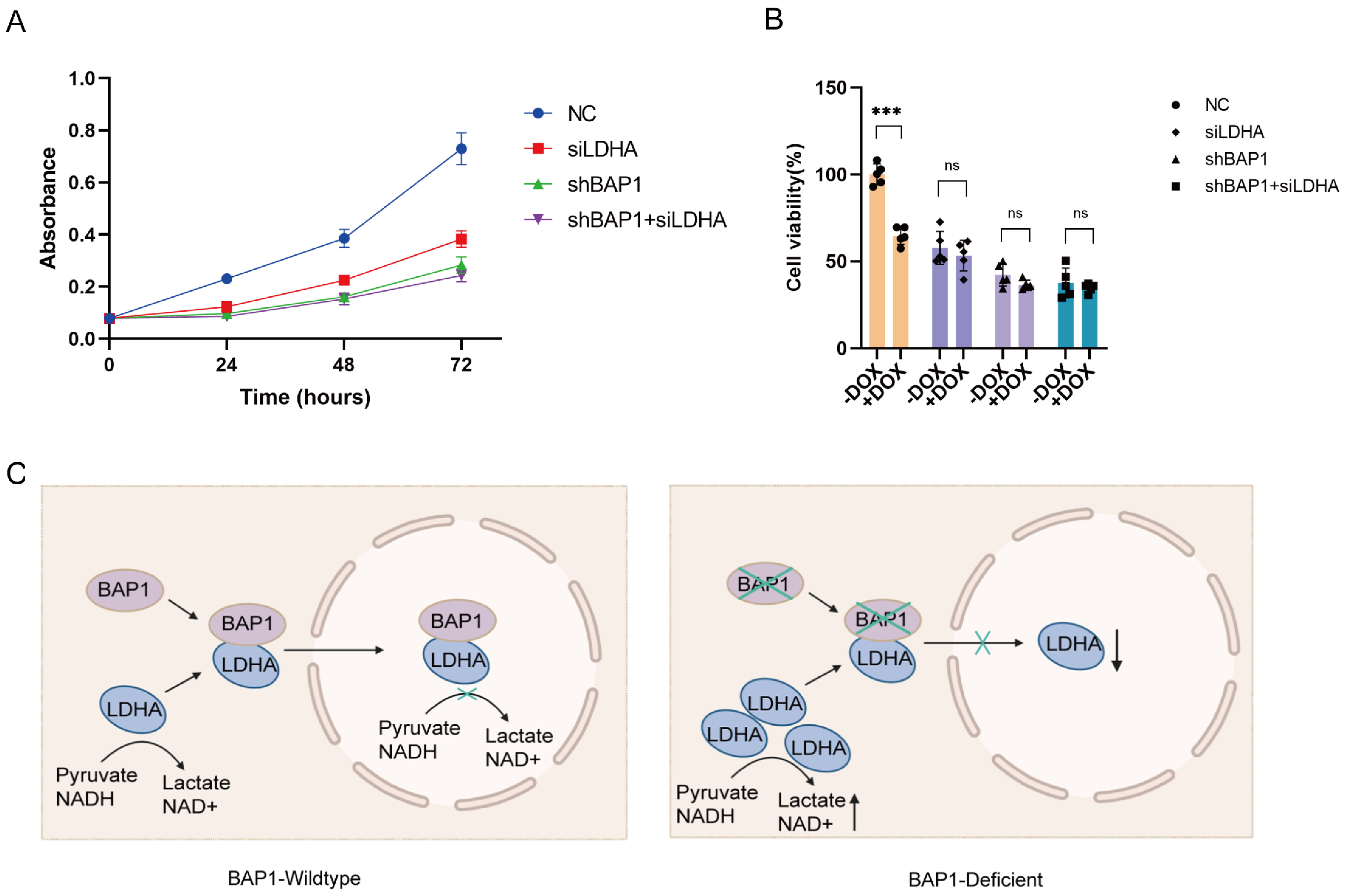

Supplement: Supplementary file 2 — Supplementary Figures [file 41420_2024_2250_MOESM2_ESM.pdf]
