# Supplementary material for: BAP1 inactivation promotes lactate production by leveraging the subcellular localization of LDHA in melanoma
Source: Cell Death Discov. 2024 Nov 26;10:483. doi: 10.1038/s41420-024-02250-6 (PMC11589756; doi:10.1038/s41420-024-02250-6)

Fig.2C

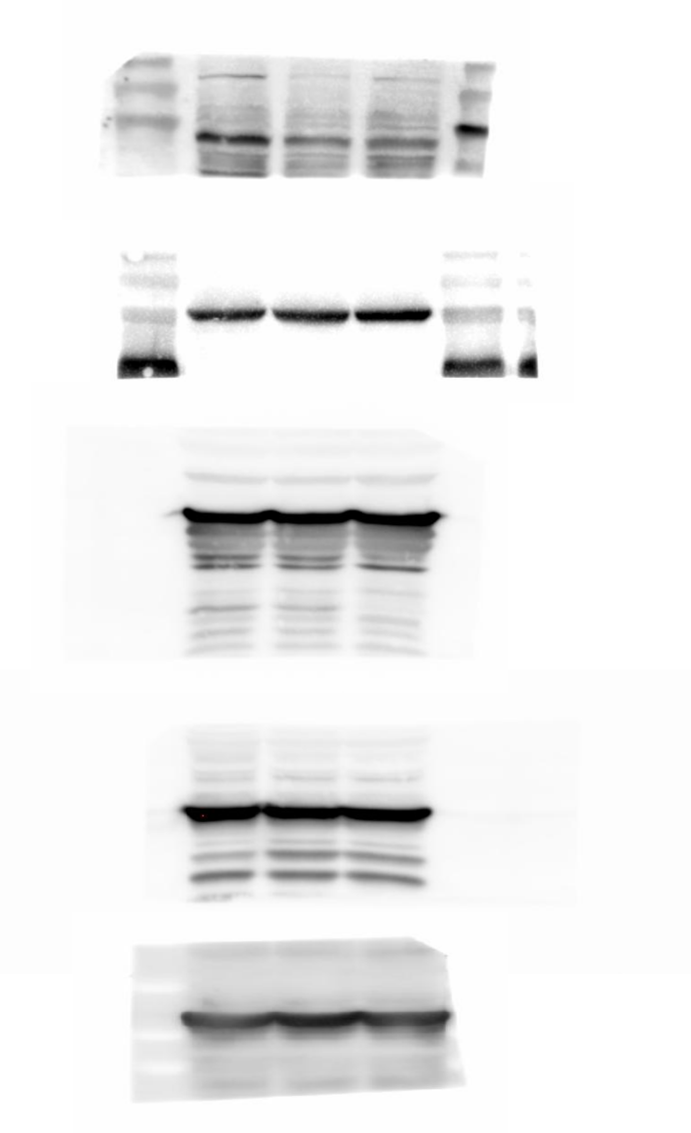

Fig.2E

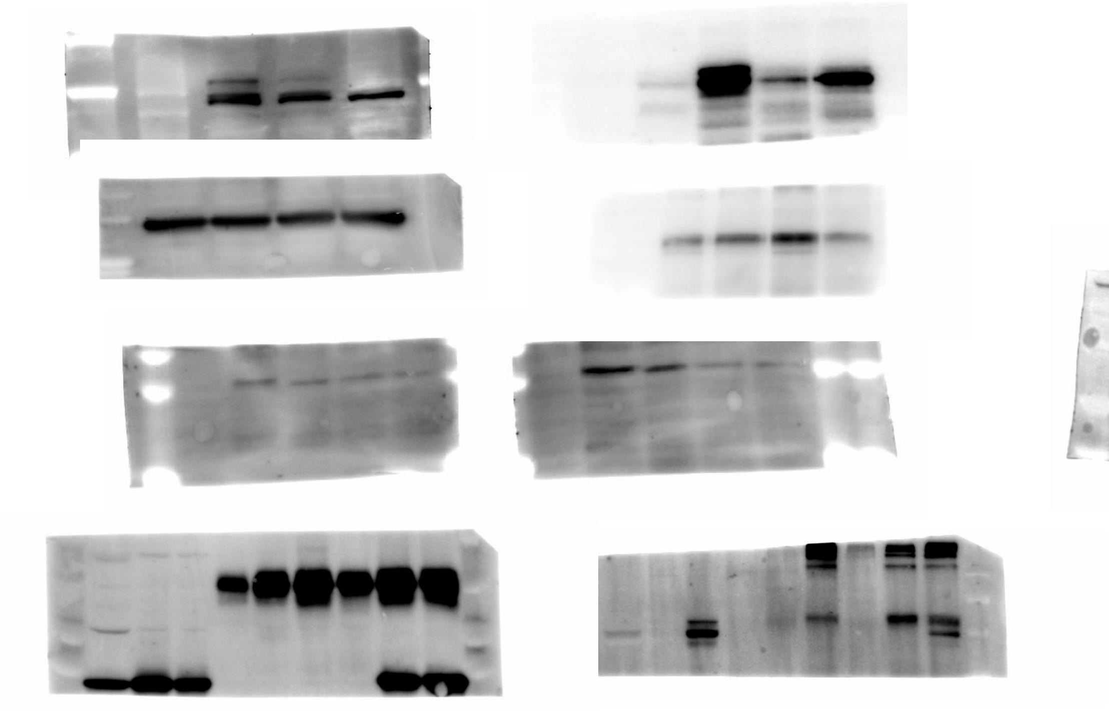

Fig.2F

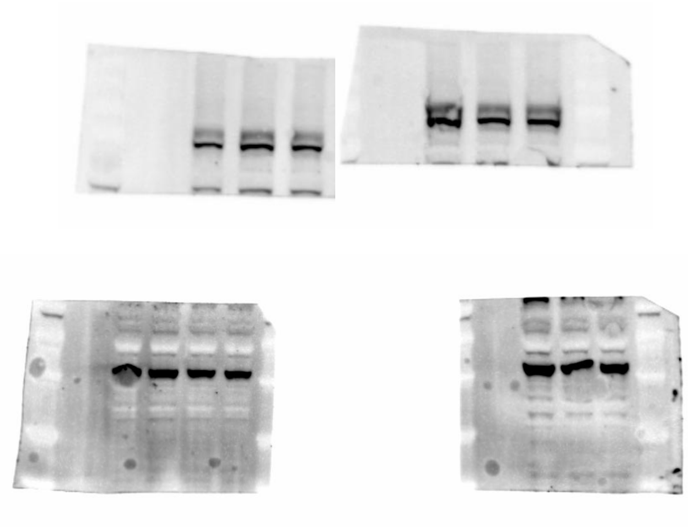

Fig.3A

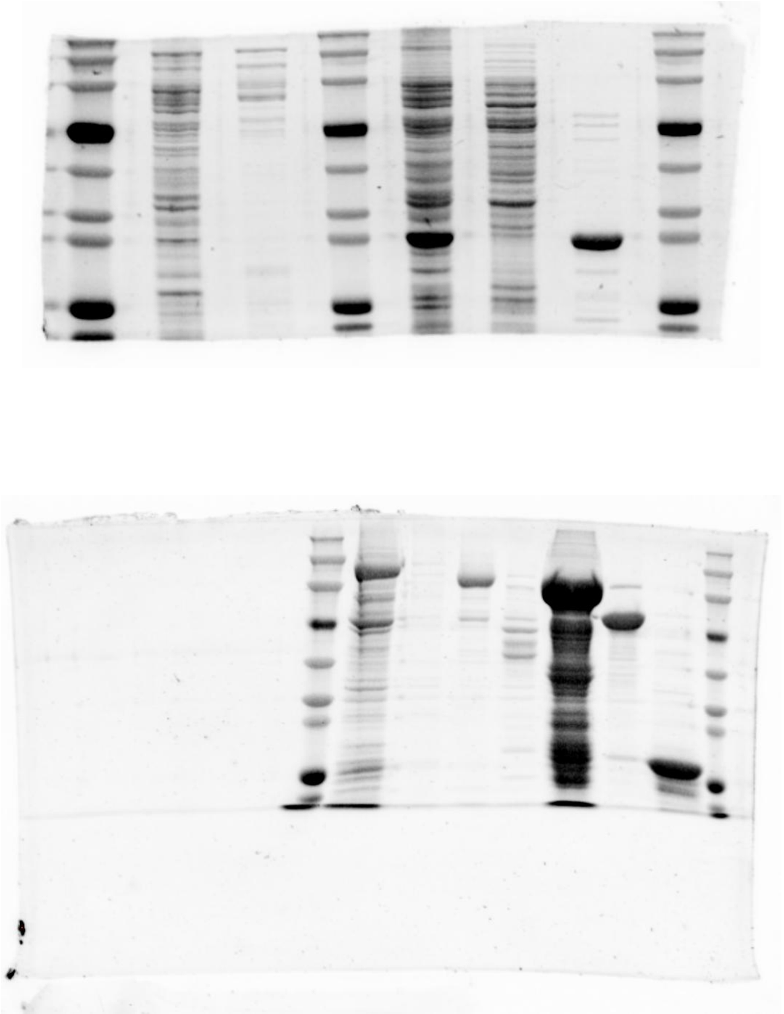

Fig.3B

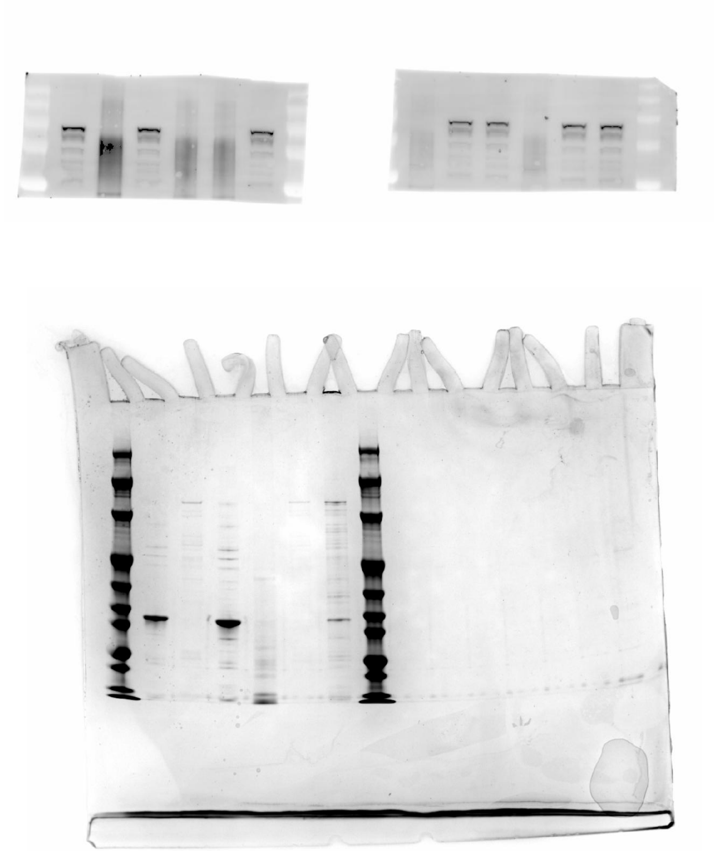

Fig.4B

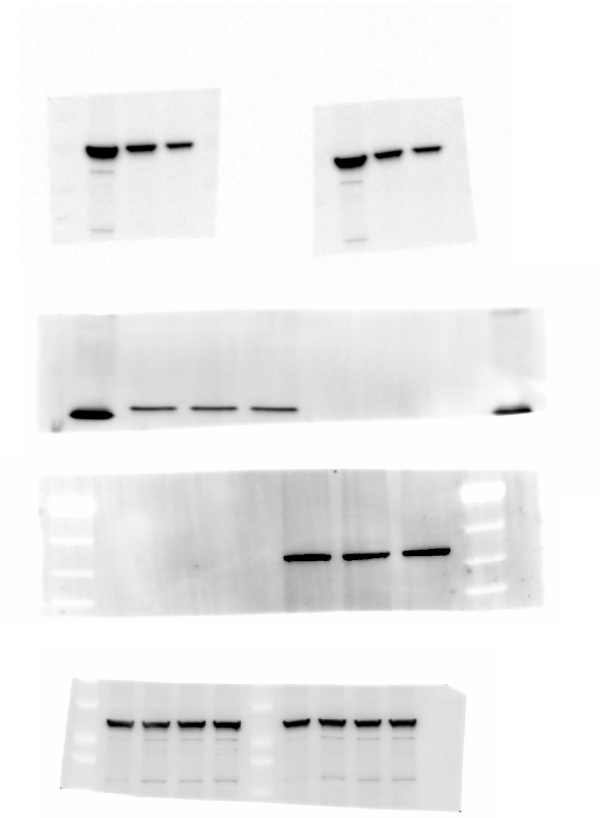

Fig.4D

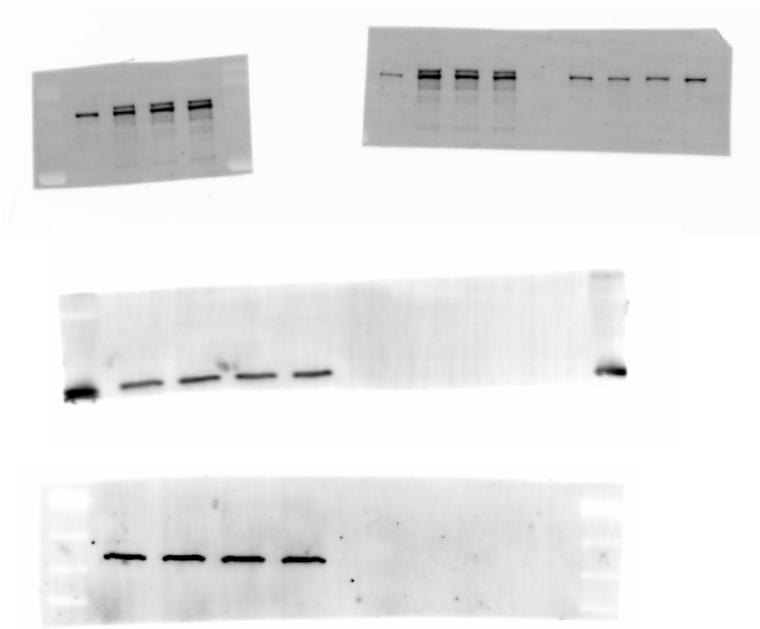

supplementary Fig.1A

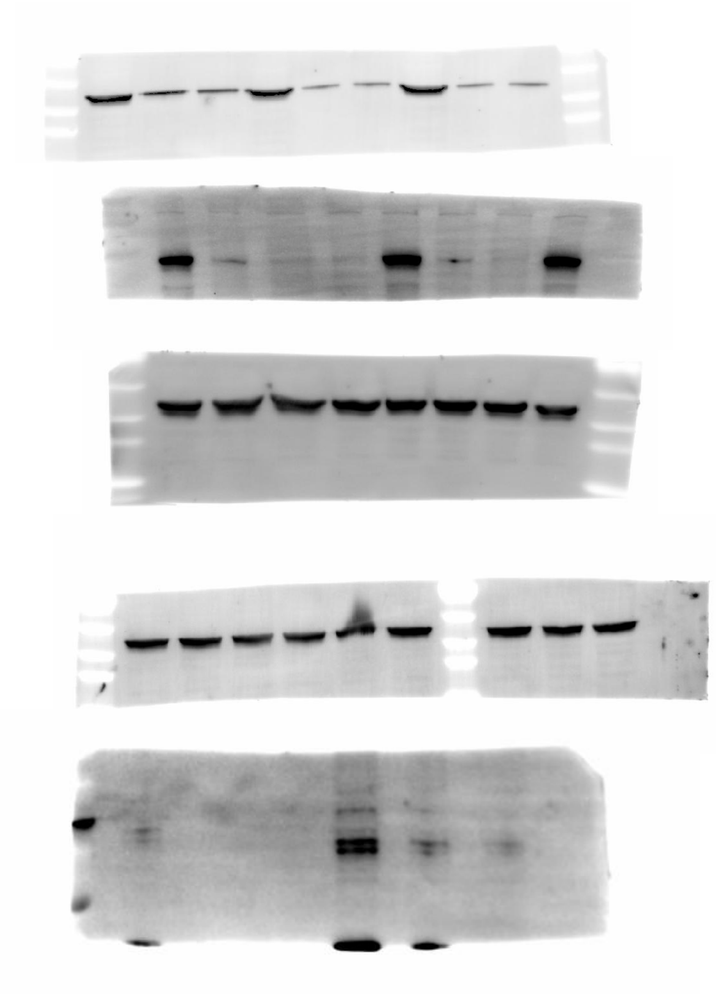

supplementary Fig.2D

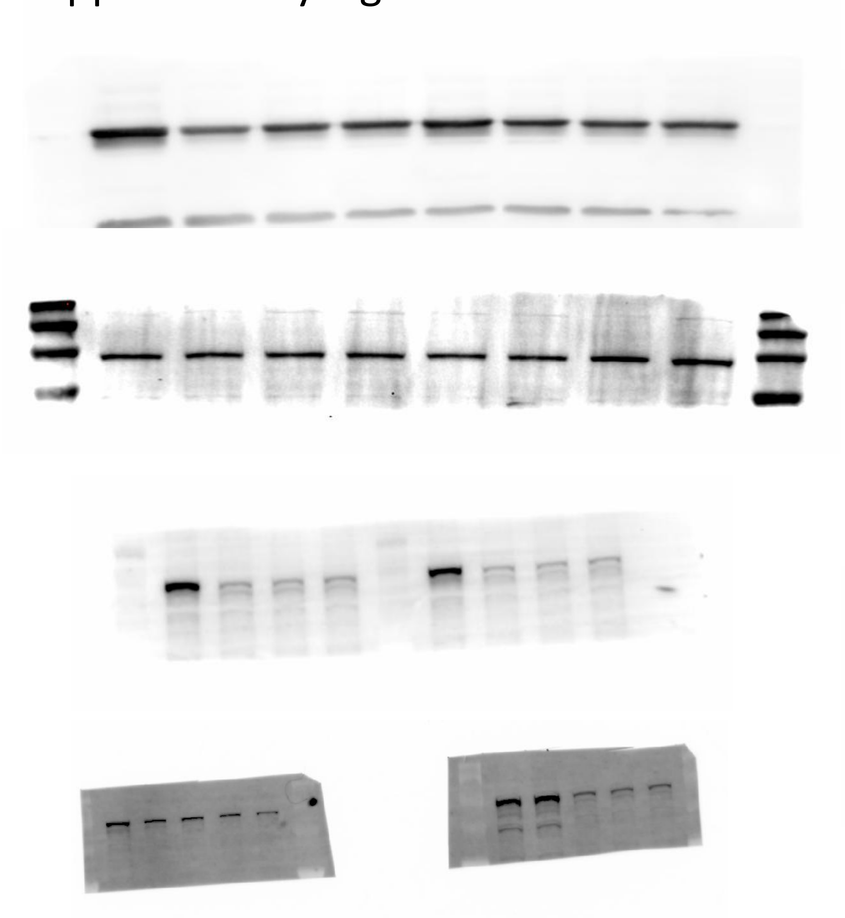

supplementary Fig.3A

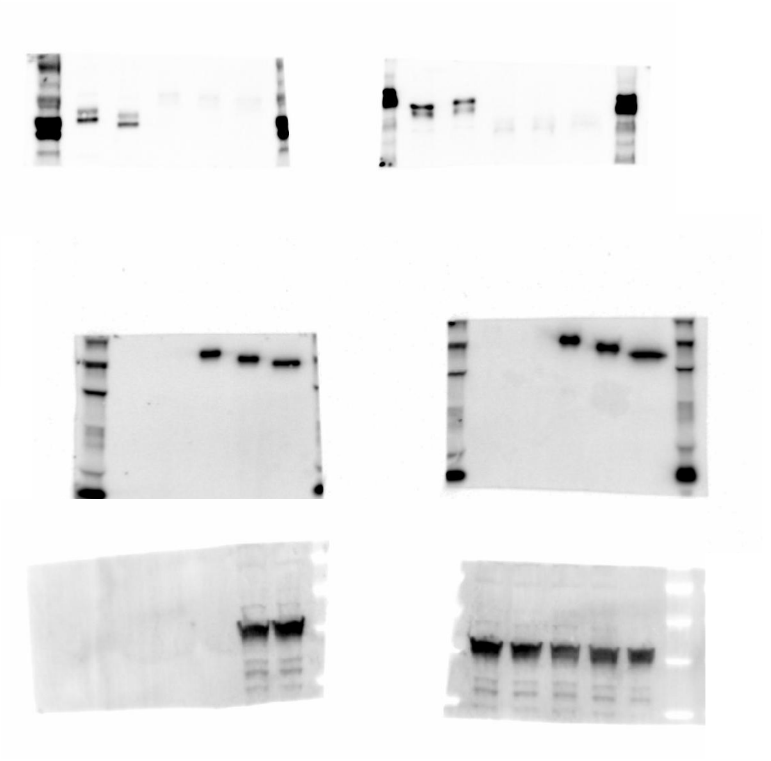

supplementary Fig.1D

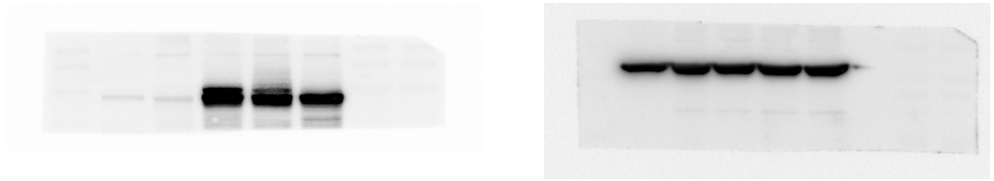

Supplement: Supplementary file 3 — WB RAW DATA [file 41420_2024_2250_MOESM3_ESM.pdf]
